# Genetic, Lipid, Fungal Microbiome and Neuroinflammatory Links Between Seborrheic Dermatitis and Parkinson’s Disease: A Narrative Review

**DOI:** 10.3390/genes17080894

**Published:** 2026-07-30

**Authors:** Vasiliki Kefala, Efthymios Oikonomou, Eleni Sfyri, Vasiliki-Sofia Grech, Niki Tertipi, Eleni Andreou, Efstathios Rallis

**Affiliations:** Department of Biomedical Sciences, School of Health and Care Sciences, University of West Attica, GR-12243 Athens, Greece; valiakef@uniwa.gr (V.K.); efoikonomou@uniwa.gr (E.O.); vgkrek@uniwa.gr (V.-S.G.); ntertipi@uniwa.gr (N.T.); elandreou@uniwa.gr (E.A.); erallis@uniwa.gr (E.R.)

**Keywords:** Parkinson’s disease, seborrheic dermatitis, alpha-synuclein, *GBA1*, *LRRK2*, *ZNF750*, *Malassezia*, ceramide, neuroinflammation, NLRP3, sebum, skin biopsy, prodromal biomarker, nerve–sebaceous gland axis

## Abstract

**Background:** Parkinson’s disease (PD) is a progressive neurodegenerative disorder with a well- documented prodromal phase during which non-motor symptoms appear years before motor onset. Seborrheic dermatitis (SD) is a chronic inflammatory skin disease that is significantly more prevalent in PD patients than in the general population. The biological mechanisms underlying this association have not been integrated into a single framework. **Objectives:** This narrative review aims to synthesise the available evidence on the molecular, genetic, and pathophysiological mechanisms linking SD and PD. **Methods:** A literature search was conducted across PubMed/MEDLINE, Scopus and ScienceDirect, supplemented by manual searches in OMIM and GeneCards. **Results:** Four major biological links between SD and PD have been proposed. First, alpha-synuclein deposits are detectable in cutaneous nerve fibres and autonomic fibres innervating sebaceous glands in PD patients and their pattern has been associated with disease subtype and progression. Second, dysfunction in ceramide and sphingolipid metabolism, associated with variants in *GBA1*, *LRRK2*, and *ZNF750*, has been linked to impaired lysosomal function and skin barrier integrity in both brain and skin tissue. Third, *Malassezia*, a commonly associated organism and possible trigger in susceptible hosts, produces metabolites that activate neuroinflammatory pathways relevant to PD and PD patients show altered *Malassezia* species composition on their skin. Fourth, NLRP3 inflammasome activation, which can be triggered by ceramide accumulation and fungal metabolites, has been proposed as a shared inflammatory mechanism that may operate in both keratinocytes and dopaminergic neurons. **Conclusions:** SD and PD appear to share overlapping genetic, lipidomic, microbial, and neuroinflammatory features. These associations are consistent with, but do not yet prove, a common pathological basis rather than a coincidental one. SD may therefore represent a potential prodromal feature or risk marker of PD. Future research should examine whether targeted intervention in high-risk SD patients can delay or modify PD onset.

## 1. Introduction

Parkinson’s disease (PD) is the second most common neurodegenerative disorder worldwide [1]. Its prevalence is increasing, and it is now a major public health problem in ageing populations. Most people recognise PD by its motor symptoms like tremor, rigidity, and slowness of movement. But the disease process begins years, sometimes decades, before any movement problem appears [2]. This period before motor onset is called the prodromal phase. During this phase, several non-motor signs can be detected, including sleep disturbances, hyposmia, constipation, and skin changes [3].

One skin condition has drawn particular attention in this context: seborrheic dermatitis (SD). SD is a chronic, relapsing inflammatory skin disease. It causes red, scaly, and greasy patches, mainly on the scalp, face, and upper chest [4]. It is important to distinguish seborrheic dermatitis from seborrhoea. Seborrhoea refers simply to excess sebum production, whereas seborrheic dermatitis is an inflammatory disease in which this sebum-rich environment, together with the yeast *Malassezia* and an altered immune response, produces visible erythema and scaling. Seborrhoea is therefore a predisposing condition rather than the disease itself: increased sebum provides the lipid substrate that *Malassezia* metabolises and the resulting inflammation gives rise to seborrheic dermatitis in exposed individuals. This distinction matters throughout the present review, because some of the changes described in PD (for example excess sebum) concern seborrhoea, while others (erythema, scaling, and *Malassezia*-driven inflammation) concern seborrheic dermatitis itself. It is common in the general population, affecting around 3–5% of adults. But in PD patients, the prevalence is much higher, with reported estimates ranging from around 18.6% to 59% depending on the population studied [5,6,7]. A large cohort study of 1.5 million veterans found that SD was significantly overrepresented in the prodromal PD group [5]. A cross-sectional study published in 2025 confirmed this association independently [6]. A systematic review and meta-analysis of dermatological manifestations in PD found SD to be one of the most consistently reported skin findings across populations [7]. Importantly, SD severity has been shown to correlate with motor symptom severity [8] and a recent review has specifically examined the clinical overlap between the two conditions [9].

Why a skin disease should be linked to a brain disease is not immediately obvious, and the explanation is unlikely to be simple. SD and PD share several biological features that suggest a deeper relationship. First, both conditions involve abnormal lipid metabolism. The sebaceous gland produces a complex mixture of lipids and its function depends on enzymes that are also relevant to PD pathogenesis [10]. Ceramides, which are central to skin barrier integrity, are also substrates of glucocerebrosidase, an enzyme encoded by the *GBA1* gene whose dysfunction is the most common genetic risk factor for PD [11]. Second, both conditions involve neuroinflammation. The NLRP3 inflammasome, a key driver of neuronal death in PD, is also activated in inflammatory skin conditions [12]. Third, both conditions are linked to *Malassezia*, a lipid-dependent yeast that colonises sebum-rich skin areas. *Malassezia* is the primary microbial trigger of SD [4] and it has been proposed as a potential contributor to neuroinflammation in PD [13]. Recent work shows that its metabolites may travel from the skin and gut to the brain [14].

The genetic architecture of PD is complex [15]. Genome-wide association studies have identified dozens of risk loci [16] and the overall genetic landscape has been reviewed in detail [17]. Several implicated genes, such as *LRRK2*, *GBA1*, *PARK2* and *SNCA*, are not only relevant to neuronal survival but also to lipid homeostasis, lysosomal function and mitochondrial integrity in peripheral tissues, including the skin. This raises the possibility that genetic vulnerability to PD may manifest in the skin before it becomes apparent in the brain.

The sebaceous gland is innervated by autonomic and sensory nerve fibres, and its secretory activity is directly regulated by the nervous system [18]. The concept of a nerve–sebaceous gland axis reflects this close anatomical and functional relationship. In PD, autonomic dysfunction is well established and there is evidence that excess sebum production in PD patients reflects peripheral autonomic denervation [19]. The composition of PD sebum is also different from that of healthy controls, with altered lipid profiles and volatile organic compounds that may be measurable non-invasively [10].

Beyond sebum, the skin offers other diagnostic opportunities. Alpha-synuclein (α-syn), which is the protein that misfolds and aggregates in PD, has been detected in cutaneous nerve fibres and sebaceous glands [20]. Skin biopsy studies have confirmed that phosphorylated α-syn deposits can be detected in PD patients with high specificity [21]. This finding has now been replicated across multiple cohorts and techniques. And skin-based seed amplification assays are emerging as clinically useful diagnostic tools [3].

## 2. Materials and Methods

### 2.1. Study Design and Research Question

This study was designed as a narrative review informed by a structured literature search. The central research question was: What are the molecular, genetic, and pathophysiological mechanisms that link the nerve–sebaceous gland axis with Parkinson’s disease, and what is the role of seborrheic dermatitis as a potential prodromal marker of the disease?

### 2.2. Literature Search Strategy

The search was conducted in April and May 2026 across three electronic databases: PubMed/MEDLINE, Scopus, and ScienceDirect. A manual search was also performed in OMIM and GeneCards for gene entries *SNCA* (#163890), *LRRK2* (#609007), *GBA* (#606463), *PARK2* (#602544), together with backward citation tracking from included review articles. Six thematic search strings were used, corresponding to the main topics of the review: (i) α-synuclein in skin and sebum; (ii) lipid and sphingolipid metabolism, including *GBA1* and *LRRK2*; (iii) *Malassezia* and seborrheic dermatitis; (iv) autonomic dysfunction and the nerve–sebaceous gland axis; (v) skin-barrier genetics, including *ZNF750* and *MPZL3*; and (vi) the NLRP3 inflammasome and neuroinflammation.

### 2.3. Study Selection and Data Management

References were managed using Rayyan (Qatar Computing Research Institute) and Zotero (v7.0). No independent second reviewer screening was performed. Consistent with the narrative (rather than systematic) design of the review, records were not enumerated in a PRISMA flow diagram, and formal counts of identified, screened, and included records were not tracked.

## 3. Results

### 3.1. Alpha-Synuclein in Skin and Sebum

#### 3.1.1. Alpha-Synuclein in Peripheral Tissues

Alpha-synuclein (α-syn) is a small protein expressed not only in the brain but also in many peripheral tissues. In PD, it misfolds and forms toxic aggregates. For a long time, research focused on what happens in the brain. But it became clear that α-syn pathology is also present in peripheral nerves, the gut, the salivary glands, and the skin [22]. This represented an important conceptual shift. If α-syn deposits can be found in accessible peripheral tissues, then diagnosis might be possible without a brain biopsy.

The skin contains a dense network of small nerve fibres, including autonomic fibres that innervate the sweat glands, blood vessels, and sebaceous glands. These fibres carry the same pathological phosphorylated form of α-syn, phospho-Ser129, that is found in the brain [23]. Alpha-synuclein is present across many peripheral tissues in PD, but skin offers a unique advantage: it is easy to sample, minimally invasive and densely innervated [20]. It should be emphasised that the α-synuclein and sebum findings discussed in Section 3.1 and Section 3.2 establish the skin and sebum as accessible biomarker sites for PD. They do not, by themselves, show that these patients have or will develop seborrheic dermatitis. These two sections are therefore best read as evidence that the skin is a useful PD biomarker compartment, which is a related but separate claim from the SD–PD link explored in Section 3.3, Section 3.4 and Section 3.5.

#### 3.1.2. Early Biopsy Evidence and Meta-Analytic Data

The first systematic evaluation of skin α-syn as a biomarker came from a meta-analysis by Tsukita et al. in 2019, which pooled data from 41 case–control studies [21]. Their analysis showed that anti-phosphorylated α-syn antibody applied to skin biopsy samples had pooled sensitivity of 0.76 and specificity of 1.00. The highest diagnostic accuracy among all peripheral tissues examined. These numbers triggered a large wave of follow-up work.

Doppler et al. had already shown in 2018 that dermal phospho-α-syn deposits were detectable in PD patients including those with *GBA* mutations, suggesting that the peripheral deposition pattern reflects the genetic background of the disease [22]. Doppler later confirmed that dermal α-syn detection remained reliable as a biomarker across different disease stages [24]. Sinclair et al. in 2021 took a complementary approach, using metabolomics of sebum to show that the lipid environment of PD skin is profoundly different from controls [25]. A finding with direct relevance to how α-syn interacts with lipid-rich surfaces such as the sebaceous gland [25].

Uehara et al. in 2021 went further and showed that RNA profiling of sebum using machine learning could distinguish PD patients from controls non-invasively [26]. This was the first demonstration that sebum, not just skin nerve fibres, carries molecular information relevant to PD diagnosis. Vacchi et al. also in 2021 used a proximity ligation assay to detect α-syn oligomers in skin, finding sensitivity of 80% and specificity of 77% for PD versus healthy controls [27]. They also documented small fibre neuropathy in PD skin, with reduced intraepidermal nerve fibre density correlating with cognitive and motor decline, suggesting that skin denervation is a marker of disease progression.

Yang et al. in 2021 examined the relationship between peripheral α-syn deposits and *LRRK2 G2385R* variants, confirming peripheral synucleinopathy in skin biopsies of genetically defined PD patients [28]. This reinforced the idea that genetic subtypes of PD share the same peripheral pathological signature.

#### 3.1.3. Deposit Patterns and Disease Subtypes

Han et al. in 2022 showed that different PD clinical subtypes have distinct α-syn deposit patterns in skin [29]. Tremor-dominant and postural instability/gait difficulty subtypes showed different distributions, raising the possibility that skin biopsy could help stratify patients clinically. Nolano et al. also in 2022 found phosphorylated α-syn in cutaneous nerves even at the earliest clinical stages of parkinsonism, supporting the use of skin biopsy as an early diagnostic tool before motor symptoms become established [30].

#### 3.1.4. Skin Biopsy in Clinical Practice and Special Populations

Donadio et al. in 2024 examined phospho-α-syn in the skin of elderly individuals without a PD diagnosis, finding deposits in some subjects and raising important questions about the specificity of the test in older populations [31]. Isaacson et al. also in 2024 studied the clinical utility of skin biopsy in a real-world tertiary care setting, finding that biopsy results changed the clinical diagnosis in 66% of cases and treatment in 55% [32]. These numbers show that skin biopsy is not just a research tool. It has direct consequences for patient management.

#### 3.1.5. Seed Amplification Assays

A major development has been the introduction of seed amplification assays (SAAs) for α-syn detection. These techniques exploit the prion-like behaviour of misfolded α-syn to amplify tiny amounts of pathological protein into a detectable signal. Kuang et al. in 2024 developed a skin-specific α-syn SAA protocol achieving high diagnostic accuracy [33]. Li et al. in 2024 validated skin-based RT-QuIC in a Chinese cohort, confirming that the technique works across different ethnic populations [34]. Wang et al. also in 2024 demonstrated that a minimally invasive skin swab, rather than a punch biopsy, could achieve sensitive and accurate PD diagnosis [35].

Braccia et al. in 2025 extended these findings to rare genetic forms of PD, showing α-syn pathology in skin nerves of a patient with an *EIF4G1* mutation [36]. Vieregge et al. in 2025 showed that dermal α-syn aggregation detected by SAA can differentiate between PD subtypes, adding a new layer of clinical utility to skin-based testing [37]. A systematic review and meta-analysis by Zhao et al. in 2025 confirmed the diagnostic value of skin α-syn assays across populations and disease stages, with pooled sensitivity and specificity both exceeding 85% for the most recent SAA-based methods [38]. Finally, Coughlin et al. in 2026 reviewed the relationship between CSF-based SAAs and skin immunofluorescence, identifying areas of convergence and outlining remaining knowledge gaps that future research needs to address [39].

Taken together, these studies establish several points that no single study demonstrates on its own. First, phosphorylated α-synuclein in cutaneous nerves is a reproducible finding across different laboratories, techniques, and ethnic populations, rather than an artefact of any one method. Second, seed amplification assays have moved skin testing from a research technique towards a clinically usable diagnostic tool, with pooled sensitivity and specificity now above 85%. Third, the density and pattern of skin deposits appear to carry information about disease subtype and progression, so the skin may serve not only as a diagnostic window but also as a potential staging tool. These are conclusions about the skin as a PD biomarker, and they do not in themselves speak to seborrheic dermatitis. These skin-based biomarker studies are summarised in Table 1.

#### 3.1.6. Cellular Mechanisms of Peripheral α-Synuclein Propagation

The detection of α-synuclein in the skin raises a further question: how does the pathology reach peripheral tissues, and how does it move between the peripheral and central nervous systems? Current evidence indicates that misfolded α-synuclein can spread from cell to cell in a prion-like manner. Several routes have been described, including release inside extracellular vesicles, transfer through tunnelling nanotubes, and receptor-mediated uptake by neighbouring cells [22,40]. Once taken up, the misfolded protein can act as a template that promotes further aggregation in the recipient cell [40].

Glial cells appear to play an important part in this process. Microglia and astrocytes can take up extracellular α-synuclein and, depending on their activation state, either help to clear it or contribute to its spread and to local inflammation [40]. In this way, neuron–glia interactions may amplify α-synuclein pathology rather than only responding to it, which links this propagation to the neuroinflammatory mechanisms discussed later in this review.

The peripheral autonomic and sensory nerves that innervate the skin, including the fibres supplying the sebaceous glands, provide a possible anatomical route for this spread [23]. Experimental and clinical observations suggest that propagation can occur in both directions: α-synuclein pathology may begin in peripheral organs and travel towards the brain (a ‘body-first’ pattern), or it may start centrally and spread outwards to peripheral nerves (a ‘brain-first’ pattern) [41]. This bidirectional model may help to explain why α-synuclein can be detected in the skin before motor symptoms appear in some patients, and it offers a mechanistic framework for viewing the nerve–sebaceous gland axis as an early site of pathology rather than a passive bystander. It should be noted, however, that much of this evidence comes from animal models and post-mortem studies, so its direct relevance to the SD–PD relationship still needs to be confirmed.

### 3.2. Lipid Metabolism, GBA1, LRRK2, and Sphingolipid Pathways

#### 3.2.1. Glucocerebrosidase and *GBA1* Mutations in Parkinson’s Disease

The *GBA1* gene encodes glucocerebrosidase (GCase), a lysosomal enzyme that breaks down glucosylceramide into ceramide and glucose. Gegg et al. in 2012 were among the first to show that GCase activity is significantly reduced in the substantia nigra of PD brains, even in patients without *GBA1* mutations [42]. This was a foundational finding. It meant that GCase dysfunction is not limited to carriers of *GBA1* variants. It is a feature of PD more broadly.

Du et al. in 2015 showed the mechanism behind this: *GBA1* deficiency promotes α-syn accumulation by blocking autophagy through inactivation of the *PPP2A* phosphatase complex [43]. Uversky in 2015 placed this in the wider context of intrinsically disordered proteins in neurodegeneration, pointing out that α-syn belongs to a class of proteins whose pathological behaviour is closely tied to their interaction with lipid membranes [44]. Mazzulli et al. in 2016 demonstrated that α-syn itself disrupts lysosomal trafficking and protein processing, creating a vicious cycle where α-syn accumulation worsens GCase dysfunction and GCase dysfunction worsens α-syn accumulation [45].

Taguchi et al. in 2017 clarified one important part of this mechanism [46]. They showed that glucosylsphingosine, a substrate that accumulates when GCase is lost, directly promotes α-syn pathology in *GBA*-associated PD models [46]. Kim et al. in 2018 then showed that inhibiting acid ceramidase, another sphingolipid-metabolising enzyme, could partially rescue α-syn accumulation in cells with *GBA1* loss of function [47]. This suggested that the sphingolipid pathway downstream of GCase is a therapeutic target, not just a passive bystander.

Ikuno et al. in 2019 used a prodromal PD mouse model to show that *GBA* haploinsufficiency accelerates α-syn pathology with concurrent changes in lipid metabolism, confirming that even a 50% reduction in GCase activity is enough to alter the lipid environment in ways that promote aggregation [48]. Cerri et al. in 2021 extended these findings to human fibroblasts, showing that *GBA* mutations influence the release and pathological cargo of small extracellular vesicles—a potential mechanism for spreading α-syn pathology from cell to cell [49].

#### 3.2.2. Clinical and Lipidomic Evidence

Guedes et al. in 2017 provided clinical evidence that *GBA*-associated PD has a distinct serum lipid profile compared to idiopathic PD and healthy controls [50]. Chan et al. also in 2017 used lipidomics to show that GM3 ganglioside, a sphingolipid, is elevated in the plasma of idiopathic PD patients, pointing to broad sphingolipid dysregulation even without *GBA1* mutations [51]. Calvano et al. in 2019 searched for lipid biomarkers in parkin-mutant skin fibroblasts using mass spectrometry, finding altered phospholipid profiles that could potentially serve as peripheral diagnostic markers [52].

Galper et al. in 2022 published two important papers in the same year [53]. The first reviewed the role of *LRRK2* in lipid pathways, covering its regulation of phosphoinositides, fatty acid metabolism, and lysosomal lipid homeostasis [53]. The second used lipidomic profiling of PD patient samples to show that lipid pathway dysfunction is prevalent across PD regardless of genetic background, affecting sphingolipids, glycerophospholipids, and cholesterol metabolism [10]. Together these papers established lipid dysregulation as a core, not peripheral, feature of PD.

Smith et al. published two complementary reviews in 2022. The first focused on *GBA* variants and the mechanisms by which they cause PD, including lysosomal dysfunction, mitochondrial impairment, and neuroinflammation [54]. The second examined the combined biochemical and clinical consequences of *GBA1* and *LRRK2* variants, showing that these two genes interact in ways that amplify disease risk [55]. Vieira et al. in 2022 provided an additional review of glucocerebrosidase mutations in PD, covering the full spectrum from mild risk variants to severe loss-of-function alleles [56].

Menozzi et al. in 2023 reviewed therapeutic strategies targeting the *GBA1* pathway, including enzyme replacement, substrate reduction therapy, and small molecule chaperones [57]. This is relevant to SD because several of these therapies also affect ceramide and sphingolipid levels in skin. Usenko et al. in 2024 examined how the *LRRK2* G2019S mutation affects lysosomal hydrolase activity, finding reduced activity of multiple lysosomal enzymes in blood cells of mutation carriers [58].

#### 3.2.3. *LRRK2* and Vesicle Trafficking

Steger et al. in 2016 used phosphoproteomics to show that LRRK2 phosphorylates a subset of Rab GTPases, which are proteins that control vesicle trafficking between cellular compartments including lysosomes [59]. This was a key mechanistic discovery. Rab GTPases regulate lipid transport, and their dysregulation by mutant *LRRK2* disrupts the delivery of lipids to lysosomes for degradation. Chang et al. in 2017 identified 17 new PD risk loci in a GWAS meta-analysis, several of which are involved in vesicle trafficking and lipid metabolism, further linking these pathways to PD risk at a population level [16]. Nalls et al. in 2019 extended this work in an even larger GWAS meta-analysis, identifying additional risk variants and providing a comprehensive view of the genetic architecture of PD risk [15]. Blauwendraat et al. in 2020 synthesised all available genetic evidence to produce the most complete picture of PD genetics at that time, confirming that lipid and lysosomal pathways are among the most enriched among PD risk genes [17].

Basharova et al. in 2025 examined the *RAB29* gene variant rs823144, showing its association with reduced lysosomal hydrolase activity in blood cells and increased PD risk [60]. RAB29 is a direct LRRK2 recruiter, and its dysfunction connects LRRK2 signalling directly to lysosomal lipid metabolism. Perez-Abshana et al. in 2025 studied the protective effect of LRRK2 kinase inhibition in human fibroblasts carrying the *GBA1* K198E variant, finding that LRRK2 inhibition partially rescued lysosomal function and reduced α-syn accumulation [61]. This supports the idea that *LRRK2* and *GBA1* operate in the same pathway and that targeting LRRK2 may benefit even non-*LRRK2* patients.

#### 3.2.4. Sphingolipids, Ceramides, and the Skin Connection

The connection between sphingolipid metabolism and skin is direct. Ceramides are the primary structural lipids of the skin barrier. Their synthesis, processing and secretion in the stratum corneum depend on many of the same enzymes, as acid ceramidase, glucocerebrosidase, sphingosine kinase, that are disrupted in PD. Wang et al. in 2025 reviewed the interplay between *GBA1*, lysosomal dysfunction and inflammation in PD, emphasising that ceramide accumulation secondary to GCase loss is not just a metabolic problem but an active driver of inflammasome activation and neuroinflammation [62].

Kumar et al. in 2025 showed that inhibiting or genetically reducing acid ceramidase (*ASAH1*) restores α-syn clearance in *GBA1*-mutant dopamine neurons derived from PD patients [63]. This finding directly links ceramide metabolism to α-syn pathology in a human cell model, and it is particularly relevant to SD because acid ceramidase activity is also abnormal in inflammatory skin conditions. Dreier et al. in 2026 showed that ambroxol, a small molecule chaperone that stabilises GCase, directly displaces α-syn from lipid membranes and prevents the formation of early protein-lipid coaggregates [64]. This suggests that the therapeutic effect of ambroxol in PD may go beyond restoring lysosomal function.

Ni et al. in 2026 applied a multi-omics approach to *GBA1*-associated PD, integrating genomics, transcriptomics, proteomics and lipidomics to show how these molecular layers interact [65]. They found that lipidomic changes, particularly in sphingolipid and glycerophospholipid species, are among the earliest and most consistent molecular signatures of *GBA1*-associated disease. Peng et al. also in 2026 specifically characterised ceramide and glycosphingolipid dysregulation in PD, confirming that these changes are not limited to the brain but are detectable in peripheral tissues and biofluids [11]. Finally, Bo RX et al. in 2022 reviewed the neuroinflammatory role of glucocerebrosidase in PD, showing how GCase loss activates microglia and promotes cytokine release through mechanisms that include ceramide accumulation and lysosomal damage [66].

Across these genetic and lipidomic studies, a single theme recurs: the enzymes and transcription factors that maintain ceramide and sphingolipid balance act in both the brain and the skin. *GBA1*, *LRRK2* and the downstream ceramide pathway are not tissue-specific, so a defect that impairs lysosomal lipid handling could in principle affect neurons and keratinocytes at the same time. What the individual studies show separately, dysfunction in one gene or one lipid class, together point towards a shared metabolic vulnerability rather than a set of unrelated observations. This remains an inference, however, because no study has yet measured these lipids in matched brain and skin from the same patients. As in Section 3.1, this evidence identifies a plausible shared pathway. It does not by itself establish that the patients concerned have seborrheic dermatitis.

### 3.3. Malassezia, Seborrheic Dermatitis Pathogenesis and the SD–PD Clinical Association

#### 3.3.1. *Malassezia* as a Commensal and Pathogen

*Malassezia* is a genus of lipid-dependent yeasts that lives on the skin of humans and most warm-blooded animals. It is part of the normal skin microbiome, but under certain conditions, it becomes pathogenic. Prohic et al. in 2016 provided a comprehensive overview of *Malassezia* species distribution in healthy skin and in dermatological conditions, describing how different species predominate in different skin areas and how shifts in species composition correlate with disease [4]. *Malassezia restricta* and *Malassezia globosa* are the species most commonly associated with SD, particularly on the scalp and face.

*Malassezia* cannot synthesise its own fatty acids. It depends entirely on host-derived lipids, which it extracts from sebum using a large arsenal of secreted lipases and phospholipases. This process damages the skin barrier, releases irritating fatty acids, and triggers immune responses. Iliev et al. in 2017 placed this in the broader context of fungal dysbiosis at mucosal and epithelial barriers, showing how commensal fungi shift from harmless colonisers to pathobionts when the host immune system or barrier function is compromised [67]. This framework is important for understanding SD: the disease is not simply caused by *Malassezia*, but by an abnormal host response to it.

Ugochukwu et al. in 2023 published two detailed reviews on the role of *Malassezia* in skin disorders [68]. The first part covered its biology, ecology, and classification as a commensal or pathogenic organism [68]. The second part focused specifically on the mechanisms by which *Malassezia* causes skin infections, including enzyme secretion, immune evasion, and biofilm formation [69]. Together, these papers provide the most thorough recent account of *Malassezia* pathobiology.

#### 3.3.2. Pathogenesis of Seborrheic Dermatitis

Wikramanayake et al. in 2019 argued that SD should be understood as a disease that goes well beyond *Malassezia* [70]. They proposed that immune dysregulation, sebaceous gland dysfunction, and genetic susceptibility all contribute independently. This view was supported by Adalsteinsson et al. in 2020, who reviewed the microbiology, immunology, and genetics of SD in detail [71]. Their review showed that SD lesions are characterised by Th1 and Th17 immune activation, elevated IL-17, IL-22, and TNF-α, and altered skin barrier function—a profile that overlaps substantially with other inflammatory skin diseases and, as we discuss later, with neuroinflammatory processes in PD.

Piacentini et al. in 2025 specifically explored the complex interplay between SD and *Malassezia* [72]. They reviewed how fungal metabolites, including indole derivatives, fatty acids, and malassezin, activate innate immune pathways and promote inflammation [72]. Turchin et al. in 2025 provided a clinical update on SD, covering its presentation, diagnosis, and management across special populations including patients with neurological diseases [73]. Khimuk in 2026 systematically reviewed the molecular mechanisms of SD, integrating recent transcriptomic and proteomic data to show how genetic susceptibility genes, including *ZNF750*, *MPZL3*, and *ACT1*, interact with microbial and environmental triggers [74]. Ungar et al. in 2025 used tape-strip profiling to characterise the immune and lipid dysregulation in SD skin non-invasively, finding a distinct molecular signature that includes downregulation of ceramide synthesis genes and the upregulation of pro-inflammatory cytokines [75].

#### 3.3.3. *Malassezia* and Parkinson’s Disease

The connection between *Malassezia* and PD was first formally proposed by Laurence et al. in 2019 [13]. They reviewed epidemiological and biological evidence suggesting that *Malassezia* could contribute to PD through several mechanisms: direct neuroinflammatory effects of fungal metabolites, molecular mimicry between fungal proteins and α-syn, and disruption of the gut–brain axis through translocation of fungal products. This hypothesis was speculative at the time but has since gained support.

Han et al. in 2023 provided the first direct microbiological evidence, showing increased diversity and altered composition of *Malassezia* species on the skin of PD patients compared to healthy controls [76]. The significance of this finding is twofold. First, it confirms that PD patients have an abnormal skin fungal microbiome. Second, it raises the question of whether this is a cause or a consequence of the disease. Does *Malassezia* overgrowth contribute to PD pathogenesis, or does the altered sebum environment of PD simply favour certain fungal species?

Naik et al. in 2025 reviewed the journey of *Malassezia* from skin and gut to the brain, detailing how fungal metabolites including indoles and fatty acid derivatives can cross epithelial barriers, enter the circulation, and reach the central nervous system [14]. They proposed that chronic low-level *Malassezia* exposure could prime the neuroinflammatory state that accelerates PD progression. Chen et al. in 2025 extended this line of inquiry to multiple system atrophy (MSA), finding alterations in the skin microbiome, including *Malassezia*, in MSA patients in a pilot study [77]. This suggests that dysbiosis of the skin fungal microbiome may be a feature of synucleinopathies more broadly, not just PD.

#### 3.3.4. The SD–PD Clinical Association

The clinical link between SD and PD has been documented in several independent studies. Tomic et al. in 2022 showed in a prospective clinical study that SD is significantly more common in PD patients and that its severity correlates with motor symptom severity, particularly rigidity and bradykinesia [8]. This correlation suggests that SD is not merely an incidental comorbidity but may reflect shared underlying pathophysiological processes.

Wilkowski et al. in 2025 confirmed this association in a cross-sectional study, finding a significantly higher prevalence of SD in PD patients compared to age- and sex-matched controls [6]. Lumbán-Ramírez et al. in 2024 reviewed the SD–PD relationship specifically from a clinical perspective, covering epidemiology, proposed mechanisms, and clinical management implications [9]. Meng et al. in 2026 took a different approach, examining bidirectional associations between SD and a range of epithelial barrier diseases in a large retrospective cohort [78]. They found that SD is associated not only with PD but with other conditions sharing epithelial barrier dysfunction, suggesting that SD sits at the intersection of barrier pathology and systemic inflammatory disease.

### 3.4. Autonomic Dysfunction, the Nerve–Sebaceous Gland Axis, and Sebum as a Biomarker

#### 3.4.1. The Neuroendocrine Control of the Sebaceous Gland

The sebaceous gland is not an autonomous structure. It is regulated by a complex network of hormonal, neural, and paracrine signals. Clayton et al. in 2020 published the most comprehensive review of the neuroendocrinology and neurobiology of sebaceous glands available in the literature [18]. They described in detail how the gland responds to androgens, neuropeptides, substance P, corticotropin-releasing hormone, and signals from the autonomic nervous system. The gland is innervated by both sympathetic and sensory fibres, and its secretory activity is directly modulated by neuronal input. This makes it a unique window into peripheral nervous system function.

In PD, this window is particularly relevant. Autonomic dysfunction is one of the most common non-motor features of the disease. It affects the heart, gut, bladder, and skin, and it can precede motor symptoms by years. Skorvanek and Bhatia in 2017 reviewed the full spectrum of skin-related manifestations in PD, including seborrheic dermatitis, sweating abnormalities, and the emerging role of skin biopsies as diagnostic tools [79]. Their review was one of the first to discuss the nerve–sebaceous gland axis explicitly in the context of PD, noting that excess sebum production in PD likely reflects autonomic denervation of sebaceous gland-innervating fibres rather than simple hormonal dysregulation.

#### 3.4.2. Sebum Hypersecretion and Its Clinical Significance

Excess sebum production, or seborrhoea, is a well-known feature of PD. It contributes directly to the development of SD in these patients. Several studies have examined why PD patients produce more sebum and whether this excess reflects a specific pathological process. Antunes et al. in 2019 highlighted the importance of recognising and managing dermatoses in parkinsonism, emphasising that skin conditions including seborrhoea are underdiagnosed and undertreated in this population [80].

Kitagawa et al. in 2024 made an important observation: they found a significant association between sebum secretion levels and cardiac sympathetic dysfunction in PD patients [81]. Patients with greater cardiac sympathetic denervation, measured by MIBG scintigraphy, had higher sebum output on the face. This suggests that sebum hypersecretion in PD is largely driven by autonomic denervation, not by androgen excess or other hormonal factors. It positions sebum secretion as a peripheral readout of sympathetic nervous system damage.

Paz and Lio in 2025 reviewed dermatological manifestations and sebum composition in PD comprehensively, covering altered lipid profiles, volatile organic compounds, and the diagnostic potential of sebum analysis [19]. They noted that the sebum of PD patients contains elevated short-chain fatty acids and disrupted sphingolipid species. Changes that are measurable non-invasively and that correlate with disease presence.

#### 3.4.3. Sebum as a Molecular Biomarker

The idea of using sebum as a liquid biopsy for PD gained momentum after a landmark study by Sinclair et al. in 2021 (already discussed in Section 3.1) which used metabolomics to identify a distinct lipid signature in PD sebum. But sebum carries more than lipids [25]. Uehara et al. in 2021 showed that sebum also contains RNA, and that machine learning applied to sebum RNA profiles can distinguish PD patients from controls with high accuracy [26]. This opened the possibility of a completely non-invasive molecular test based on a simple skin swab.

Dinesh and colleagues in 2021 examined skin conditions in early PD, documenting that seborrhoea and SD are already present at the time of first clinical presentation, often having been present for years before the patient sought neurological help [82]. Niemann et al. in 2021 provided a broad review of PD and skin, covering the full range of skin manifestations and their pathophysiological basis, and situating sebum changes within the wider context of autonomic and neurological dysfunction [3].

#### 3.4.4. Skin Fibroblasts as a Model for PD

Skin fibroblasts have become an important experimental model for studying PD at the cellular level. They are obtained easily from a skin punch biopsy, they carry the same genetic mutations as neurons, and they can be maintained in culture for extended periods. González-Casacuberta et al. in 2019 used fibroblasts from PD patients with *PARK2* mutations to characterise mitochondrial and autophagic dysfunction, finding impaired mitophagy and altered mitochondrial morphology [83]. Zuev et al. in 2019 explored the potential of skin fibroblasts as a clinical diagnostic tool for PD, examining whether cellular phenotypes in fibroblasts from PD patients of different ages could serve as biomarkers [84].

#### 3.4.5. Prodromal Skin Changes and Epidemiological Context

Two large epidemiological studies provide essential context for this section. Scott et al. in 2021 analysed data from 1.5 million veterans and found that SD was significantly associated with prodromal PD, appearing years before motor diagnosis [5]. This was the largest study to date to examine skin conditions as prodromal PD markers. Schrag et al. in 2023 widened the spectrum further, analysing a broad range of risk factors and prodromal features of PD in a large population-based cohort [2]. They confirmed that skin conditions, autonomic symptoms, and sensory disturbances cluster in the years before PD diagnosis, painting a picture of a systemic prodrome that begins in peripheral tissues long before dopaminergic neurons show clinical signs of failure.

Yalçınkaya Iyidal et al. in 2024 examined the prevalence and clinical significance of skin manifestations specifically in PD patients, finding that SD, seborrhoea, and related conditions were present in a high proportion of the cohort and that their severity reflected overall disease burden [85]. Djuanda et al. in 2026 conducted a systematic review and single-arm meta-analysis of dermatological manifestations in PD, providing pooled prevalence estimates for SD and other skin conditions across multiple populations and confirming that SD is the most consistently reported skin finding in PD [7].

### 3.5. Skin Barrier Dysfunction, ZNF750, and Shared Neuroinflammatory Pathways

#### 3.5.1. The Genetic Basis of Seborrheic Dermatitis

SD has long been considered a disease without a clear genetic foundation, but this view has changed. Karakadze et al. in 2018 conducted the first systematic review of the genetic basis of SD, identifying 11 gene mutations or protein deficiencies that cause SD or an SD-like phenotype in humans and experimental animals [86]. The implicated genes fall into two broad groups: those involved in immune regulation, including *ACT1*, *C5*, *IKBKG/NEMO*, and *STK4* and those involved in epidermal differentiation and barrier formation, including *ZNF750* and *MPZL3*. This genetic map is important because it shows that SD is not simply a response to *Malassezia* but has intrinsic biological determinants that make certain individuals susceptible.

Khimuk in 2026 extended this analysis using more recent transcriptomic and proteomic data, building a more detailed molecular picture of SD pathogenesis [74]. This review confirmed that the same genetic pathways identified by Karakadze et al. remain central, but also highlighted new candidate genes and signalling networks, including JAK-STAT pathways, toll-like receptor signalling, and lipid metabolic regulators, that may be amenable to therapeutic targeting.

#### 3.5.2. *ZNF750* and the Skin Barrier

ZNF750 is a transcription factor that controls the terminal differentiation of keratinocytes and the formation of the skin barrier. Loss-of-function variants in *ZNF750* cause an SD-like phenotype with scaling, erythema, and barrier disruption. Butera et al. in 2023 showed that ZNF750 (referred to as ZFP750 in their study) regulates lipid metabolism in the skin, specifically controlling the expression of genes involved in ceramide synthesis and fatty acid elongation [87]. When *ZNF750* function is lost, ceramide chain composition shifts, barrier integrity fails, and the skin becomes susceptible to microbial dysbiosis and inflammation.

This is directly relevant to the SD–PD connection. The same ceramide species that are disrupted by *ZNF750* loss are also altered in PD through *GBA1* dysfunction. Rousel et al. in 2024 confirmed this in a clinical study of SD patients, showing that lesional skin has a perturbed ceramide subclass composition with impaired chain elongation and increased chain unsaturation [88]. Importantly, barrier impairment correlated strongly with ceramide composition changes, suggesting that the two are not independent but mechanistically linked. Ungar et al. in 2025, whose tape-strip profiling data were discussed in Section 3.3, also found downregulation of ceramide synthesis genes in SD lesional skin, consistent with *ZNF750*-driven dysregulation [75].

Van Smeden et al. in 2020 provided important mechanistic context by studying skin barrier lipid enzyme activity in Netherton syndrome patients, a rare genetic skin disease with severe barrier dysfunction [89]. They showed that abnormal ceramide composition results directly from altered lipid-processing enzyme activity and that this creates a self-reinforcing cycle of barrier failure and inflammation. The same principle applies to SD, where reduced GCase activity in the skin, mirroring what happens in the brain in PD, may be one mechanism driving ceramide abnormalities. These shared genetic and molecular links between SD and PD are summarised in Table 2.

#### 3.5.3. Neuroinflammation as a Shared Pathway

The most important bridge between SD and PD at the molecular level may be neuroinflammation. Heneka et al. in 2014 established the framework for understanding innate immune activation in neurodegeneration, showing how pattern recognition receptors, cytokine cascades, and microglial activation contribute to the progression of diseases including PD [1]. This framework applies not only to the brain but to peripheral tissues where innate immune cells, including skin macrophages and dendritic cells, respond to the same molecular signals.

Gordon et al. in 2018 showed that inflammasome inhibition prevents α-syn pathology and dopaminergic neurodegeneration in mice, establishing a direct causal link between inflammasome activation and PD progression [90]. The inflammasome they identified was NLRP3, the same complex that is activated in inflammatory skin conditions. Lauterbach et al. in 2021 provided a mechanistic link between sphingolipid metabolism and NLRP3 activation, showing that 1-deoxysphingolipids, aberrant sphingolipid species that accumulate when sphingolipid metabolism is disrupted, cause autophagosome and lysosome accumulation and directly trigger NLRP3 inflammasome activation [91]. This is particularly relevant because 1-deoxysphingolipids are elevated in conditions with GCase dysfunction, connecting the lipid pathway directly to inflammasome biology.

Xu et al. in 2025 reviewed the role of the NLRP3 inflammasome in neuroinflammation and central nervous system diseases comprehensively, covering its activation mechanisms, its role in α-syn-driven microglia activation, and its contribution to dopaminergic neuron death in PD [12]. They also discussed emerging therapeutic approaches targeting NLRP3, including small molecule inhibitors and biological agents, some of which are already in clinical trials for other inflammatory conditions.

Bo RX et al. in 2022 reviewed the neuroinflammatory role of glucocerebrosidase in PD specifically, showing how GCase loss activates microglia through ceramide accumulation, lysosomal damage, and the release of damage-associated molecular patterns [66]. This paper closes the loop between the lipid pathway, the skin barrier, and neuroinflammation: GCase dysfunction disrupts ceramide metabolism in both skin and brain, triggers NLRP3 activation in both compartments, and drives inflammatory cascades that are pathologically similar whether they occur in keratinocytes or in microglia.

#### 3.5.4. Shared Inflammatory Signatures Between SD and PD

The inflammatory profile of SD skin lesions, elevated IL-1β, IL-17, IL-22, TNF-α, and activated NLRP3, is strikingly similar to the neuroinflammatory profile documented in PD brain tissue and cerebrospinal fluid. This parallel has not been studied directly in human SD–PD patients, but the convergence of the molecular evidence is compelling. Van Smeden et al.’s work on ceramide enzymes [89], Lauterbach et al.’s work on 1-deoxysphingolipids and NLRP3 [91], Gordon et al.’s inflammasome inhibition data [90], and Xu et al.’s comprehensive NLRP3 review [12] together suggest a model in which ceramide dysregulation, driven by GCase and *ZNF750* dysfunction, activates NLRP3 in both skin and brain, creating a shared inflammatory state that manifests as SD peripherally and as neurodegeneration centrally.

This model is consistent with the epidemiological data reviewed in earlier sections: SD precedes PD diagnosis, correlates with motor severity, and tracks with autonomic dysfunction. It is also consistent with the genetic data: *GBA1* mutations affect ceramide metabolism in both skin and brain, and *ZNF750* loss disrupts ceramide synthesis specifically in the skin in a way that mirrors *GBA1*-driven disruption in neurons.

## 4. Discussion

This review has brought together evidence from five thematic domains (α-synuclein pathology, lipid and sphingolipid metabolism, the *Malassezia* microbiome, autonomic control of the sebaceous gland, and skin-barrier and neuroinflammatory pathways) that converge on four main proposed mechanistic links between SD and PD. Much of this evidence is associative rather than causal. It spans genetics, lipidomics, microbiology, immunology and clinical epidemiology and it points towards a small number of candidate shared mechanisms rather than a proven common cause.

The most consistent thread running through all five sections is lipid metabolism—specifically ceramide and sphingolipid homeostasis. *GBA1* dysfunction disrupts ceramide metabolism in the lysosome. *ZNF750* dysfunction disrupts ceramide synthesis in the skin barrier. *LRRK2* mutations disrupt lipid transport between cellular compartments. The end result in all three cases is an abnormal accumulation of ceramide precursors, a failure of normal lipid processing, and a downstream activation of inflammatory cascades including the *NLRP3* inflammasome. That the same molecular disruption occurs in dopaminergic neurons in the brain and in keratinocytes and sebaceous gland cells in the skin is unlikely to be entirely coincidental. Because ceramide metabolism is a fundamental process shared by many tissues, a defect in it could plausibly affect both the brain and skin, although this simultaneous involvement has not yet been demonstrated directly.

The second thread is α-syn. The skin is now established as a site of early α-syn pathology. Phosphorylated α-syn deposits appear in cutaneous nerve fibres before motor symptoms are clinically evident. They can be detected by immunofluorescence, proximity ligation assay, and seed amplification assays. Their pattern has been associated with PD subtype. Their density correlates with disease progression. The sebaceous gland, richly innervated by the same autonomic fibres that carry α-syn pathology, sits at the centre of this picture. Sebum hypersecretion in PD has been linked to autonomic denervation of the nerve–sebaceous gland axis. Altered sebum composition may reflect the same lipid dysregulation implicated in neurodegeneration in the brain.

The third thread is *Malassezia*. Its role in SD is well established. Its potential role in PD is more speculative but increasingly supported. The observations that PD patients have an altered skin *Malassezia* microbiome, that *Malassezia* metabolites can reach the brain, and that fungal-derived indoles and fatty acids activate the same neuroinflammatory pathways are implicated in PD progression. Taken together, these findings justify a serious investigation of *Malassezia* as a contributor to PD pathogenesis rather than just a marker of skin disease.

### 4.1. Strengths and Limitations

This review has several strengths. It is the first narrative review to integrate the genetic, lipidomic, fungal microbiome, and neuroinflammatory dimensions of the SD–PD relationship in a single framework. It draws exclusively on primary research and reviews published in peer-reviewed journals. The search strategy was structured and reproducible, using six thematic search strings across three databases with manual supplementation from OMIM and GeneCards.

Two recent reviews have addressed related ground. Lumbán-Ramírez et al. (2024) examined the SD–PD relationship from a mainly clinical perspective, covering epidemiology, proposed mechanisms, and management [9]. Paz and Lio (2025) reviewed dermatological manifestations and sebum composition in PD [19]. The present review differs from both in three specific ways. First, it integrates the genetic and lipidomic evidence (*GBA1*, *LRRK2*, *ZNF750*, *MPZL3* and the ceramide/sphingolipid pathway) with the clinical and microbial evidence, instead of treating these as separate topics. Second, it places the *NLRP3* inflammasome at the centre as a candidate shared downstream mechanism operating in both keratinocytes and dopaminergic neurons, which neither earlier review develops. Third, it explicitly separates seborrhoea, seborrheic dermatitis, and skin-based PD biomarkers as distinct concepts, and it frames the proposed *Malassezia* mechanisms by their strength of evidence (Table 3) rather than presenting all links as equally established.

There are also important limitations. This is a narrative review, not a systematic one. The selection of papers involved judgement at multiple stages and is therefore subject to selection bias. The thematic categorisation of papers into five axes reflects the authors’ interpretive framework, and other groupings are possible. Many of the mechanistic papers included in this review used cell lines or animal models and their direct relevance to human disease and specifically to the SD–PD relationship, must be inferred rather than proven. No study in this review directly tested the hypothesis that SD and PD share a common molecular cause in the same patient cohort at the tissue level. That study has not yet been done.

Another limitation is that the direction of causality in the SD–PD association remains unclear. SD could be a prodromal manifestation of the same pathological process that causes PD, or it could be a consequence of autonomic denervation that occurs as PD progresses, or both. The epidemiological evidence, particularly the finding that SD precedes PD diagnosis by years, favours the prodromal hypothesis. But this cannot be confirmed without longitudinal molecular studies in pre-motor PD cohorts.

A further limitation is that the SD–PD association may be explained in part by factors other than a shared molecular pathway. Dopaminergic and other medications can alter sebum production. Motor impairment and reduced ability can lead to poorer facial hygiene, which may worsen or unmask SD. And because PD patients are under closer medical observation, skin findings may simply be recorded more often (ascertainment bias). These confounders do not exclude a shared-pathology model, but they are plausible alternative explanations that future studies should measure and control for.

### 4.2. Clinical Implications

If the model proposed in this review is correct, several clinical implications follow. First, SD should be considered a potential prodromal feature/risk marker of PD. Dermatologists who regularly see patients with SD, particularly middle-aged or elderly patients with severe or treatment-resistant SD, should be aware of the association. A history of SD in a patient presenting with early motor symptoms, hyposmia, or REM sleep behaviour disorder should increase clinical suspicion for PD.

Second, sebum analysis has real potential as a non-invasive diagnostic tool for PD. Metabolomic profiling of sebum, RNA profiling of sebum, and volatile organic compound analysis of skin secretions are all technically feasible and have shown promising results. They are far less invasive than lumbar puncture or dopamine transporter imaging, and they could in principle be deployed in primary care or dermatology clinics. What is needed now is validation in large prospective cohorts with longitudinal follow-up.

Third, skin biopsy for α-syn detection is no longer a research curiosity. It is a clinically validated procedure that changes diagnosis and treatment in a majority of patients who undergo it. Its integration into the diagnostic pathway for atypical parkinsonism and early PD should be considered. The move toward skin swab-based SAAs, if validated further, would make this even more accessible.

Fourth, the shared lipid and inflammatory pathways between SD and PD open therapeutic possibilities. Agents that restore GCase activity, such as ambroxol, or that modulate ceramide metabolism may have effects in both the brain and the skin. *NLRP3* inhibitors, which are currently in clinical development for several inflammatory diseases, could in principle be tested in SD patients with genetic PD risk. These are speculative proposals, but they are biologically grounded.

### 4.3. Future Research Directions

Several specific research questions emerge from this review.

The most important is whether SD patients with *GBA1*, *LRRK2*, or *SNCA* variants show earlier or more severe skin pathology than genetically unaffected SD patients, and whether their skin contains detectable α-syn deposits. This question can now be answered with existing tools, such as genetic testing, skin biopsy, and SAA, in a prospective cohort design.

A second question is whether *Malassezia* species composition on PD skin correlates with neuroinflammatory markers in cerebrospinal fluid or blood. If fungal dysbiosis on the skin reflects or drives systemic inflammation, this relationship should be measurable. A third question is whether treating SD aggressively in patients at high PD risk, using antifungal agents, barrier-restoring topicals, or ceramide supplementation, has any effect on PD biomarkers or disease onset. This is a high-risk hypothesis, but one that is now biologically justifiable.

Finally, multi-omics studies integrating genomics, transcriptomics, proteomics, and lipidomics in matched skin and brain tissue from the same PD patients, similar to what Ni et al. did in 2026 for *GBA1*-associated PD, are needed to test whether the molecular convergence proposed in this review is real or apparent.

## 5. Conclusions

Seborrheic dermatitis and Parkinson’s disease appear to be connected at several levels. They may share genetic and molecular pathways, including *GBA1*-related lysosomal ceramide dysregulation and *ZNF750*-related barrier lipid dysregulation. They appear to share an association with *Malassezia*, whose products can activate similar innate immune pathways in skin and brain. They share a molecular marker, α-syn, that appears in the cutaneous nerves of the sebaceous gland axis before PD is clinically diagnosed. Finally, they may share an inflammatory mechanism: *NLRP3* inflammasome activation, linked to ceramide dysregulation, that has been described in both keratinocytes and dopaminergic neurons.

The available evidence suggests that the skin is not merely a passive site in Parkinson’s disease but may be an active participant in its pathology. The nerve–sebaceous gland axis is both a site of early pathology and a source of accessible biomarkers. Sebum, skin nerve fibres, and fibroblasts all carry molecular information that can be sampled non-invasively or with minimal invasion. The tools to exploit this information, such as SAAs, metabolomics, RNA profiling and skin biopsy are available now.

What is needed is a shift in perspective. SD should not be regarded only as a cosmetic concern in PD patients. It may instead represent a window into the disease and potentially a cutaneous reflection of some of the same molecular dysfunction that affects neurons in the substantia nigra. With that recognition comes the possibility of earlier diagnosis, better stratification, and eventually, intervention at a stage when the disease is still modifiable.

## Figures and Tables

**Table 1:** Skin-based biomarkers for the diagnosis of Parkinson’s disease.

Method	Sample	Sensitivity	Specificity	Study (Year)	n	Comment
Anti-p-alpha-syn immunofluorescence (skin biopsy)	Punch biopsy (3 mm); multiple sites	0.76 (pooled)	1.00 (pooled)	Tsukita et al. (2019) [21]	41 studies pooled	Highest specificity among all peripheral alpha-syn biopsy sites; gold-standard reference for comparison
Proximity ligation assay (PLA)—alpha-syn oligomers	Punch biopsy	80%	77%	Vacchi et al. (2021) [27]	75	Detects early toxic oligomeric species; also reveals small fibre neuropathy correlating with motor/cognitive decline
RT-QuIC (skin-based seed amplification assay)	Punch biopsy	93.3%	100%	Li et al. (2024) [34]	58	Validated in Chinese cohort; confirms cross-ethnic applicability of SAA technique
Skin-specific alpha-syn SAA (optimised protocol)	Punch biopsy	High (kappa 0.87)	High	Kuang et al. (2024) [33]	617	Largest skin SAA cohort; validated across 150 clinical conditions; subtype differentiation possible
Minimally invasive skin swab (alpha-syn)	Skin surface swab (non-biopsy)	High	High	Wang et al. (2024) [35]	Not stated	First proof-of-concept for non-biopsy alpha-syn detection; potential for primary care deployment
Dermal alpha-syn SAA (PD subtype differentiation)	Punch biopsy	High	High	Vieregge et al. (2025) [37]	Multicenter	SAA parameters differentiate tremor-dominant from PIGD subtypes; supports personalised diagnostic use
Skin alpha-syn assays—pooled meta-analysis (SAA + IHC)	Punch biopsy	>85% (pooled)	>85% (pooled)	Zhao et al. (2025) [38]	Multiple cohorts	Most recent meta-analysis; SAA-based methods outperform IHC; confirms clinical utility across populations
Sebum metabolomics (LC-MS lipid profiling)	Sebum swab	Not reported	Not reported	Sinclair et al. (2021) [25]	274	Identifies distinct PD sebum lipid signature; short-chain fatty acids and sphingolipid species altered; Nature Communications
Sebum RNA profiling with machine learning	Sebum swab	AUC 0.806	AUC 0.806	Uehara et al. (2021) [26]	130 (double cohort)	First demonstration that sebum RNA carries PD diagnostic signal; completely non-invasive; Scientific Reports
Skin biopsy—clinical utility (real-world setting)	Punch biopsy	N/A	N/A	Isaacson et al. (2024) [32]	97	Retrospective study; biopsy changed diagnosis in 66% and treatment in 55%; demonstrates direct clinical impact

Studies are listed in order of methodology (biopsy-based alpha-syn detection first, then minimally invasive and sebum-based approaches). AUC = area under the receiver operating characteristic curve; IHC = immunohistochemistry; LC-MS = liquid chromatography mass spectrometry; n = total participants; PIGD = postural instability and gait difficulty; RT-QuIC = real-time quaking-induced conversion; SAA = seed amplification assay.

**Table 2:** Shared genetic and molecular links between seborrheic dermatitis and Parkinson’s disease.

Gene/Protein	Role in PD Pathogenesis	Role in SD/Skin	Shared Mechanism	Key References
*GBA1/GCase*	Encodes lysosomal glucocerebrosidase; loss-of-function variants are the most common genetic PD risk factor; GCase deficiency impairs alpha-syn clearance and drives ceramide accumulation	GCase activity is required for normal stratum corneum ceramide processing; reduced GCase activity in skin disrupts ceramide chain composition and impairs barrier integrity	Ceramide/sphingolipid dysregulation in both brain (neurodegeneration) and skin (barrier failure); NLRP3 inflammasome activation downstream of ceramide accumulation in both tissues	[42,43,44,45,46,47,48,49,54,55,56,57,62,63,87,89,90,91]
*LRRK2*	Encodes a Rab GTPase kinase; *G2019S* and *G2385R* mutations are among most common familial PD causes; dysregulates vesicle trafficking, lysosomal lipid delivery, and alpha-syn pathology	Regulates vesicle-mediated lipid transport in keratinocytes; interacts with *GBA1* pathway; LRRK2 kinase inhibition rescues lysosomal function in *GBA1*-mutant skin fibroblasts	Lysosomal lipid transport failure and Rab GTPase dysregulation in both neurons and skin cells; *LRRK2* and *GBA1* operate in the same pathway amplifying ceramide and alpha-syn pathology	[28,45,53,58,59,60,61,65,67]
*ZNF750*	Not directly implicated in PD neurodegeneration; however, ZNF750 regulates ceramide synthesis genes that overlap with *GBA1*-driven ceramide pathways relevant to PD	Keratinocyte differentiation transcription factor; loss-of-function variants cause an SD-like phenotype with scaling, erythema, and shifts in ceramide composition	Disruption of ceramide synthesis in skin mirrors *GBA1*-driven ceramide disruption in brain; converges on NLRP3 activation and a shared inflammatory cytokine profile (IL-1beta, IL-17, TNF-alpha)	[74,75,86,87,88]
*MPZL3*	Not directly implicated in PD neurodegeneration	Susceptibility gene for SD; encodes a regulator of epidermal differentiation and lipid/barrier function in keratinocytes	Contributes to skin-barrier and lipid dysregulation in SD; included as an SD susceptibility gene rather than a direct PD link	[74,86]
*SNCA (alpha-synuclein)*	Encodes alpha-synuclein; mutations and multiplications cause familial PD; phospho-alpha-syn aggregates in Lewy bodies are the hallmark of PD pathology	Phospho-alpha-syn deposits are detected in sebaceous gland-innervating cutaneous autonomic nerve fibres in PD patients; deposit pattern predicts PD subtype and correlates with SD severity	Cutaneous alpha-syn pathology in the nerve–sebaceous gland axis provides a mechanistic link between neurodegeneration and SD; sebum lipid environment modulates alpha-syn membrane interactions	[22,23,24,25,26,27,28,29,30,31,32,33,34,35,36,37,38,39]
*PARK2 (Parkin)*	E3 ubiquitin ligase; loss-of-function mutations cause early-onset autosomal recessive PD; critical for mitophagy and mitochondrial quality control	*PARK2*-mutant skin fibroblasts show impaired mitophagy and altered phospholipid profiles; fibroblasts serve as a peripheral cellular model of PD-related lipid dysfunction	Mitochondrial dysfunction and abnormal lipid metabolism in skin fibroblasts mirror neuronal pathology; shared ceramide and phospholipid abnormalities link peripheral (skin) and central (brain) compartments	[20,52,83,84]

Gene/protein entries are listed in order of discussion in the text. Refs = reference numbers as cited in the main text.

**Table 3:** *Malassezia*: proposed mechanisms linking seborrheic dermatitis to Parkinson’s disease.

Proposed Mechanism	Evidence	Key Study (Year)	Evidence Type	Strength
Lipase/phospholipase-driven skin barrier disruption	*Malassezia* secretes lipases and phospholipases that hydrolyse host sebum triglycerides, releasing irritating unsaturated fatty acids and oleic acid; this disrupts stratum corneum ceramide composition and triggers Th1/Th17 inflammation—the same pathway documented in SD and partly overlapping with *GBA1*-driven ceramide disruption in PD	Prohic et al. (2016) [4]; Adalsteinsson et al. (2020) [71]; Rousel et al. (2024) [88]	In vitro/clinical	Strong
Activation of innate immune pathways (TLR/NLRP3)	*Malassezia* cell wall components—beta-glucans, mannans, and phospholipomannan—activate TLR2, TLR4, and Dectin-1 on keratinocytes and dendritic cells, triggering NF-kB and downstream *NLRP3* inflammasome activation; this mirrors the *NLRP3*-driven neuroinflammation that drives dopaminergic neuron loss in PD	Adalsteinsson et al. (2020) [71]; Piacentini et al. (2025) [72]; Xu et al. (2025) [12]	Experimental/review	Moderate
Indole derivative production and neuroactive metabolites	*Malassezia* produces indole-3-acetic acid, malassezin, and pityriacitrin—tryptophan-derived indoles that are aryl hydrocarbon receptor (AhR) agonists; AhR activation modulates neuroinflammation and dopaminergic neuron survival; indoles can cross epithelial barriers and potentially the blood–brain barrier	Naik et al. (2025) [14]; Piacentini et al. (2025) [72]; Laurence et al. (2019) [13]	Review/in vitro	Indirect
Altered *Malassezia* species composition on PD skin	Nested PCR of sebum samples from PD patients showed *Malassezia slooffiae* OR 9.358 vs. controls; increased overall *Malassezia* species diversity on PD skin versus age- and sex-matched controls; whether this reflects altered sebum substrate availability or active pathobiont selection is unclear	Han et al. (2023) [76]	Case–control microbiome	Moderate
*Malassezia* microbiome dysbiosis in other synucleinopathies (MSA)	Pilot case–control study found altered skin fungal microbiome composition including *Malassezia* in multiple system atrophy patients; suggests that skin fungal dysbiosis may be a shared feature of synucleinopathies rather than specific to PD	Chen et al. (2025) [77]	Pilot case–control	Indirect
Molecular mimicry between *Malassezia* proteins and alpha-synuclein	Structural homology between certain *Malassezia* surface proteins and the C-terminal region of alpha-synuclein has been proposed; chronic immune exposure to *Malassezia* antigens could theoretically prime autoreactive T cells that cross-react with alpha-syn peptides, contributing to neuroinflammation	Laurence et al. (2019) [13]	Hypothesis/bioinformatics	Speculative
Gut-skin-brain axis: translocation of *Malassezia* products	*Malassezia* has been detected in gut samples; fungal metabolites including beta-glucans and indoles can translocate across gut epithelium, enter portal circulation, and reach systemic sites including the CNS; this gut-skin-brain route may amplify peripheral fungal signals into central neuroinflammatory responses	Naik et al. (2025) [14]; Iliev and Leonardi (2017) [67]	Review/translational	Indirect

Evidence strength graded as: Strong = direct experimental or clinical evidence in human studies; Moderate = indirect human evidence or validated animal/cell models; Indirect = plausible mechanism supported by analogous data; Speculative = hypothesis based on structural or bioinformatic analysis only. AhR = aryl hydrocarbon receptor; CNS = central nervous system; MSA = multiple system atrophy; OR = odds ratio; TLR = toll-like receptor.

## Data Availability

No new data were created or analysed in this study. Data sharing is not applicable to this article.

## Abbreviations

The following abbreviations are used in this manuscript:

PD	Parkinson’s disease
SD	Seborrheic dermatitis
α-syn	Alpha-synuclein
GBA1	Glucosylceramidase beta 1 gene
LRRK2	Leucine-rich repeat kinase 2 gene
ZNF750	Zinc finger protein 750 gene
NLRP3	NLR family pyrin domain-containing protein 3
SNCA	Synuclein alpha gene
GWAS	Genome-wide association study
PARK2	Parkin RBR E3 ubiquitin protein ligase gene
OMIM	Online Mendelian Inheritance in Man
SAA	Seed amplification assay
RT-QuIC	Real-time quaking-induced conversion
PLA	Proximity ligation assay
IF	Immunofluorescence
IHC	Immunohistochemistry
MSA	Multiple system atrophy
CSF	Cerebrospinal fluid
GCase	Glucocerebrosidase enzyme
GlcSph	Glucosylsphingosine
ASAH1	Acid ceramidase 1 gene (N-acylsphingosine amidohydrolase 1)
EV	Extracellular vesicle
iPSC	Induced pluripotent stem cell
LC-MS	Liquid chromatography mass spectrometry
AUC	Area under the receiver operating characteristic curve
TLR	Toll-like receptor
NF-κB	Nuclear factor kappa-light-chain-enhancer of activated B cells
IL-17	Interleukin-17
IL-22	Interleukin-22
TNF-α	Tumour necrosis factor alpha
IL-1β	Interleukin-1 beta
AhR	Aryl hydrocarbon receptor
CNS	Central nervous system
MIBG	Meta-iodobenzylguanidine (cardiac sympathetic scintigraphy)
VOC	Volatile organic compound
PIGD	Postural instability and gait difficulty
REM	Rapid eye movement (REM sleep behaviour disorder)
H/M	Heart-to-mediastinum ratio (MIBG scintigraphy)
JAK-STAT	Janus kinase–signal transducer and activator of transcription pathway
MPZL3	Myelin protein zero-like 3 gene
ACT1	Adaptor protein for interleukin-17 receptor signalling
IKBKG	Inhibitor of nuclear factor kappa-B kinase subunit gamma gene
STK4	Serine/threonine kinase 4 gene
OR	Odds ratio
PPP2A	Protein phosphatase 2A
RAB29	Ras-related protein Rab-29 gene
DAMP	Damage-associated molecular pattern
UPDRS	Unified Parkinson’s Disease Rating Scale
SASI	Seborrheic Area and Severity Index
NGS	Next-generation sequencing
PCR	Polymerase chain reaction
HILIC	Hydrophilic interaction liquid chromatography
ESI-MS	Electrospray ionisation mass spectrometry
